# Enamel Resistance to Demineralization After Bracket Debonding Using Fluoride Varnish

**DOI:** 10.1038/s41598-017-15600-5

**Published:** 2017-11-09

**Authors:** Ascensión Vicente, Antonio José Ortiz Ruiz, Miriam García López, Yolanda Martínez Beneyto, Luis-Alberto Bravo-González

**Affiliations:** 1 Department of Orthodontics, Faculty of Medicine, University of Murcia, Hospital Morales Meseguer, 2ª planta, C/Marqués de los Vélez s/n, 30008 Murcia, Spain; 2 Department of Integral Pediatric Dentistry, Faculty of Medicine, University of Murcia, Hospital Morales Meseguer, 2ª planta, C/Marqués de los Vélez s/n, 30008 Murcia, Spain; 3 Department of Preventive Dentistry, Faculty of Medicine, University of Murcia, Hospital Morales Meseguer, 2ª planta, C/Marqués de los Vélez s/n, 30008 Murcia, Spain

## Abstract

The aim of this study was to evaluate the elemental content and morphology of enamel subjected to demineralization cycles after bracket debonding, adhesive remnant removal, and application of a fluoride varnish. 125 bovine teeth were divided into five groups (n = 25): 1) Intact enamel; 2) Intact enamel + demineralization cycles (DC); 3) Enamel after adhesive removal; 4)Enamel after adhesive removal + DC; 5) Enamel after adhesive removal + Profluorid + DC. The weight percentages of calcium (Ca), phosphorous (P) and fluoride (F) were calculated using energy dispersive X-ray spectroscopy (EDX). Samples were observed under scanning electron microscopy (SEM). Data were analyzed using Kruskal-Wallis and Mann-Whitney test. The weight percentages of Ca and P in Group 1 were significantly higher than Groups 2, 4, and 5. The weight percentages of Ca and P in Group 2 were significantly higher than Groups 4 and 5. Group 3 presented significantly higher percentages of Ca and P than Group 4. Group 5 showed a significantly higher percentage of Ca than Group 4. The presence of F was detected in Group 5. SEM images showed more signs of demineralization in Group 4 than Group 5. Fluoride varnish application may protect enamel from demineralization after bracket debonding.

## Introduction

Bracket debonding is a frequent and unavoidable event in orthodontic treatment. Brackets may debond due to adhesive failure, or be removed deliberately by the orthodontist to correct bracket position or when treatment comes to an end^1,2^.

In the bracket bonding and debonding process, enamel loss is mainly due to the polishing performed before acid etching, the acid etch itself, and to the removal of adhesive remaining on the dental surface after debonding, being the procedure of removal of the adhesive which produces more surface loss^3^. On the other hand, the act of debonding can also produce enamel loss. Pon *et al*.^4^ detected the presence of calcium (%) on the bases of debonded brackets, the percentage being higher when the quantity of adhesive remaining on the bracket base was greater.

According to some authors, the loss of dental material resulting from bracket removal and tooth surface cleaning reaches a depth of 20–30 microns^5^, while others claim that it represents a loss of some 50 microns^6^. In any case, it is clear that the surface layer of enamel – the most resistant to acid dissolution – is lost as a result of removing orthodontic apparatus, leaving enamel that is more susceptible to demineralization in the oral medium^7^.

It has been shown that the use of fluoride varnishes contributes to a reduction in the incidence of caries, and so its application is recommended as a public health measure^8,9^. In orthodontics, these varnishes have been used to prevent white spot lesions during treatments involving brackets^10^, to resolve the lesions when they appear after treatment^11,12^ and following interproximal enamel reduction^13,14^. However, as far as we are aware, no research has been published on the application of fluoride varnish after bracket removal as a preventative measure against demineralization.

This study aimed to evaluate the elemental content and morphology of enamel subjected to demineralization cycles after bracket debonding, adhesive removal, and fluoride varnish application.

## Materials and Methods

### Sample preparation

The study used 125 bovine incisors, which were washed in distilled water and submerged in 0.1% thymol solution for 24 hours. Afterwards, the teeth were placed in distilled water, which was changed every 24 hours until the moment of use to avoid deterioration.

The vestibular surfaces of all teeth were cleaned by brushing with prophylactic cream (Detartrine, Septodont, France), washed, and dried with compressed air.

Seventy-five upper central incisor brackets (Victory Series®, 3M Unitek Dental Products, Monrovia, California, USA) were bonded to the vestibular surfaces of 75 teeth with Transbond Plus Self Etching Primer (3M Unitek Dental Products, Monrovia, California, USA) and Transbond XT Paste (3M Unitek Dental Products, Monrovia, California, USA) following the manufacturer’s instructions for each product. Excess resin around the bracket base was removed with a dental probe. Brackets were polymerized for 10 seconds on each side of the bracket with the SmartLite LED lamp (Dentsply^®^, USA) at 1250 W/cm^2^. The curing distance was 0 mm (surface contact).

Afterwards the teeth were covered with an acid-resistant nail varnish (60 Seconds nail varnish, Rimmel, London, UK) leaving 2 mm of enamel uncovered around each bracket. The 50 teeth that did not receive brackets were also covered in nail varnish leaving an area of exposed enamel of 2 × 4 mm.

Samples were stored in saliva for 24 hours at 37 °C, then bracket removal was performed using debonding pliers (678–219 0914, Hu-Friedy, Germany). Remaining adhesive material was removed from the enamel using a carbide tungsten bur (T21XR, Edenta Ag, Switzerland), followed by polishing with Soft-Lex discs (3M ESPE Soft-Lex tm. Dental Products, St Paul, USA). Adhesive removal was considered complete when the material was no longer visible under the light of the operatory lamp.

### Experimental groups

The 125 teeth were divided into five groups (n = 25): Group 1) Intact enamel; Group 2) Intact enamel + demineralization cycles (DC); Group 3) Enamel after adhesive removal; Group 4) Enamel after adhesive removal + DC; Group 5) Enamel after adhesive removal + Profluorid + DC.

### Varnish application

Profluorid^®^ Varnish (VOCO GmbH. Cuxhaven, Germany) was applied to the enamel surfaces in Group 5, which had been cleaned and dried previously, following the manufacturers’ instructions. The varnish was left to dry for one minute before being placed in artificial saliva. Table 1 details the composition of Profluorid^®^ Varnish.

**Table 1:** Product composition according material safety data sheets (MSDS).

Varnish	Composition	% by Wt
VOCO Profluorid varnish	Ethanol/Sodium fluoride	10–25/2.5–5

### Sample storage and demineralization cycles

Groups 1 and 3 were kept in artificial saliva at 37° for 8 days. The saliva composition used as storage medium was: 1% carmellose sodium, 13% sorbitol, 0.12% potassium chloride, 0.084% sodium chloride, 0.005% magnesium chloride hexahydrate, 0.015% calcium chloride anhydrous, 0.017% potassium phosphate dibasic, and 0.1% Nigapin® sodium. The saliva pH was adjusted and maintained at 6.57^15^.

Group 2, 4 and 5 samples were submerged in artificial saliva at 37 °C for 8 days, and subjected to demineralization cycles as follows: samples were placed in a demineralizing solution for 2 hours, three times per day, and returned to artificial saliva between the 2-hour cycles. Samples were washed in distilled water at each change of medium. The demineralization solution and the artificial saliva were changed every 48 hours^14^.

The composition of the demineralization solution was as follows: 2.2 mM calcium chloride (CaCl_2_ 2H_2_O); 2.2 mM monosodium phosphate (NaH_2_PO_4 7_H_2_O); 0.05 mM lactic acid; pH was adjusted to 4.5 with 50% sodium hydroxide (NaOH)^16^.

### Sample preparation for EDX/SEM analysis

Tooth surfaces in all groups were washed in distilled water and Group 5 samples were cleaned with a dental prophylaxis brush to eliminate the varnish before quantifying the enamel elements and examining the enamel morphology. All teeth were sectioned at the cement-enamel junction using a diamond disc (Komet Dental, Gebr. Brasseler GmbH & Co. Lemgo. Germany). Then, all crowns were given an ultrasonic bath for 60 minutes at room temperature in order to eliminate any remaining varnish from the surfaces, or any other impurity that might interfere with EDX and SEM observation.

#### EDX analysis

Twenty surfaces in each group were coated with carbon and analyzed using a JEOL-6100 scanning electron microscope (Jeol Ltd., Tokyo, Japan) equipped with an INCA energy dispersive X-ray microanalysis system (Oxford Instruments Analytical, Oxfordshire, U.K.) at 20 KV, with a counting time of 100 s per source.

The elements quantified were: Ca (weight %), P (weight %) and F (weight %). Using the Ca and P values obtained, Ca/P stoichiometric ratios were calculated using the following formula: Ca (mol)/P (mol) % = [Ca (weight %)/40.08 (g/mol)]/[P (weight %)/30.97 (g/mol)], the molecular masses of Ca and P being 40.08 and 30.97 respectively.

#### SEM analysis

Five samples from each group were coated with gold and examined under ×1000 magnification at 20 KV. The most representative images were captured and stored.

### Statistical analysis

Statistical analysis was performed using the SPSS 19.0 statistical software package (IBM SPSS Inc., New York, USA).

Weight percentage values for Ca and P, as well as Ca/P stoichiometric ratios underwent the Kolmogorov-Smirnov normal distribution test (p < 0.05) and the Levene test for homogeneity of variance (p < 0.05). As they did not fulfill normality criteria (p > 0.05) or homogeneity of variance (p > 0.05), data were analyzed using the Kruskal-Wallis test (p < 0.05) and the Mann-Whitney test applying Bonferroni Correction (p < 0.005).

## Results

### EDX

Results of EDX analysis are shown in Table 2.

**Table 2:** Weight percentage (mean ± standard deviation) of calcium (Ca), phosphorous (P) and Fluor (F). Ratio Ca/P (mol/mol).

Groups	Ca	P	Ca/P	F
1) Intact enamel	50.69 ± 4.52	19.76 ± 0.62	1.98 ± 0.15	0
2) Intact enamel + DC	46.81 ± 2.13*	18.77 ± 1.19*	1.93 ± 0.10	0
3) Enamel after adhesive removal	48.54 ± 18.04	19.01 ± 0.83	1.88 ± 0.26	0
4) Enamel after adhesive removal + DC	29.95 ± 5.43*^†#^	15.12 ± 2.01*^†#^	1.52 ± 0.10*^†^	0
5) Enamel after adhesive removal + Proflourid + DC	34.99 ± 3.45*^†¶^	17.3 ± 2.49*^†^	1.59 ± 0.22*^†^	0.31 ± 0.11

DC: demineralization cycles. The Mann-Whitney test applying Bonferroni correction (p < 0.005) found significant differences between groups: *vs Intact enamel. ^†^vs Intact enamel + DC. ^#^ vs Enamel after adhesive removal. ^¶^vs Enamel after adhesive removal +DC.

The Kruskal Wallis test found significant differences in the weight percentage of Ca (p = 0.00) and P (p = 0.00), as well as in the Ca/P stoichiometric ratios (p = 0.00) between different groups.

The Mann Whitney test found that the percentage weight of Ca and P in Group 1 (Intact enamel) was significantly higher than in Group 2 (Intact enamel + DC; p = 0.000 and p = 0.003 respectively), Group 4 (Enamel after adhesive removal + DC; p = 0.000) and Group 5 (Enamel after adhesive removal + Profluorid + DC; p = 0.000).

For Group 2 (Intact enamel + DC), the percentage weight of Ca and P was significantly higher than Group 4 (Enamel after adhesive removal + DC; p = 0.000) and Group 5 (Enamel after adhesive removal + Profluorid + DC; p = 0.000).

Group 3 (Enamel after adhesive removal group) showed significantly higher Ca and P weight percentages than Group 4 (Enamel after adhesive removal + DC group; p = 0.000 and p = 0.002, respectively).

At the same time, Group 5 (Enamel after adhesive removal + Profluorid + DC) showed a significantly higher Ca percentage weight than Group 4 (Enamel after adhesive removal + DC; p = 0.000).

No statistically significant differences were found in Ca and P weight percentages (p > 0,005) in the rest of the comparisons between groups.

As for Ca/P stoichiometric ratios, the Mann Whitney test found that Groups 1 and 2 (Intact enamel and Intact enamel + DC) showed significantly higher ratios than Groups 4 (Enamel after adhesive removal + DC; p = 0.000) and 5 (Enamel after adhesive removal + Profluorid + DC; p = 0.000). No statistically significant differences (p > 0.005) were identified in comparisons between the other groups.

The presence of F (%) was only detected in Group 5 (Enamel after adhesive removal + Profluorid + DC); F was found in all the teeth in Group 5 with a mean value and standard deviation of 0.31 ± 0.11. (Table 2)

### SEM

**Figure 1:**
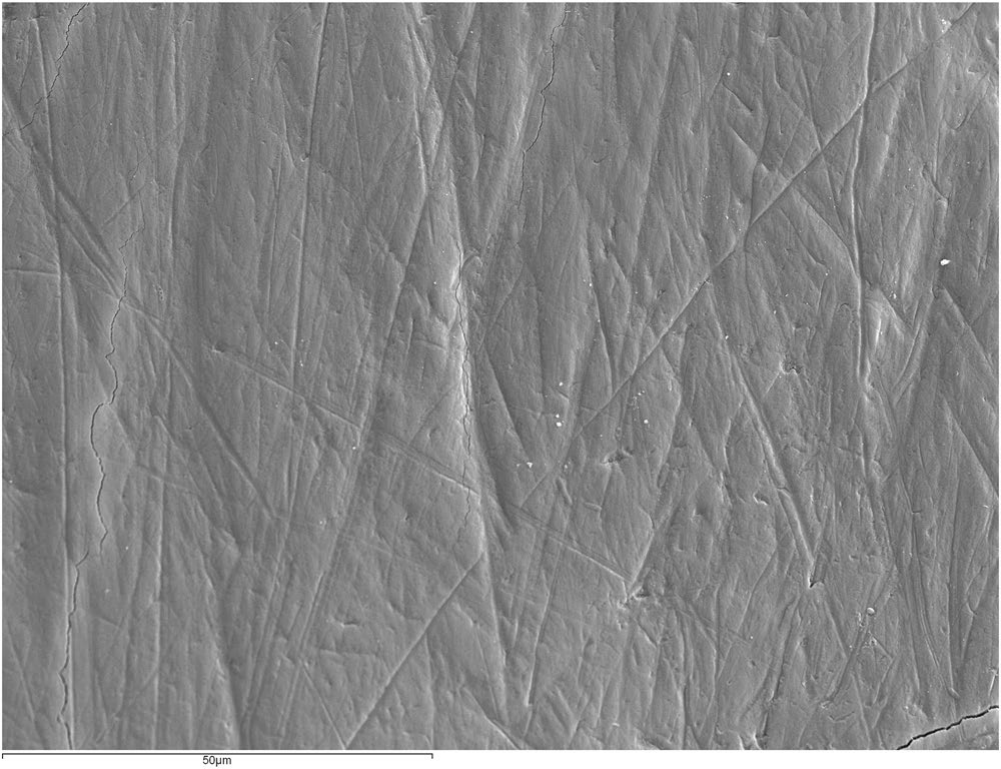
Group 1: Intact enamel. SEM micrograph (x1000).

**Figure 2:**
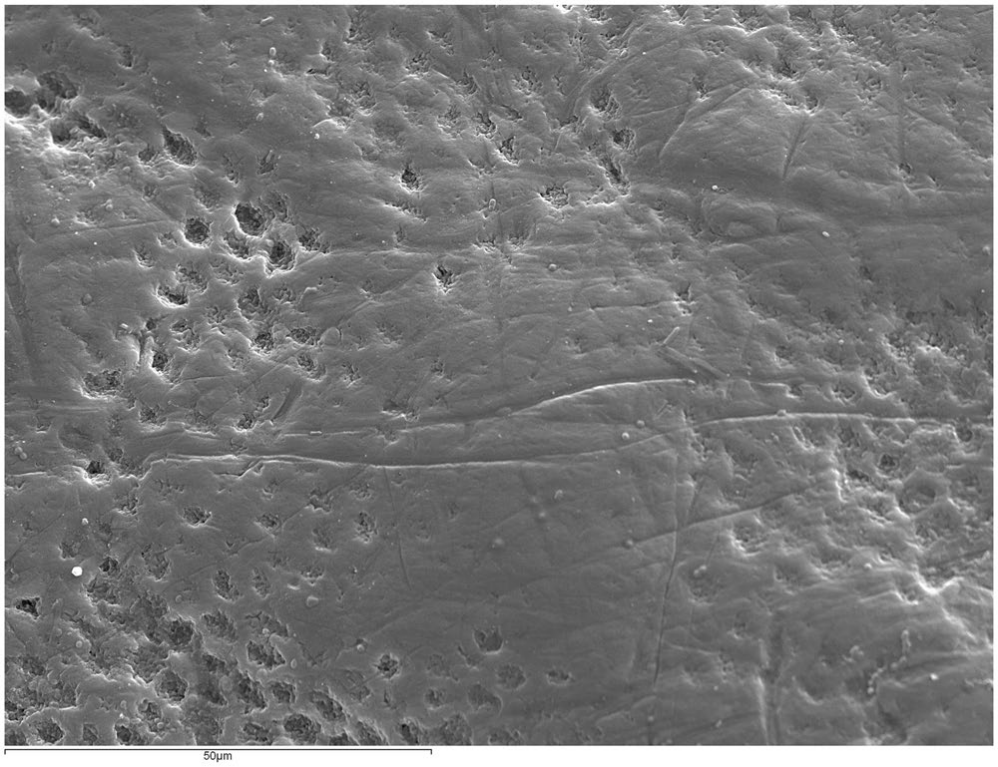
Group 2: Intact enamel + DC. SEM micrograph (x1000).

**Figure 3:**
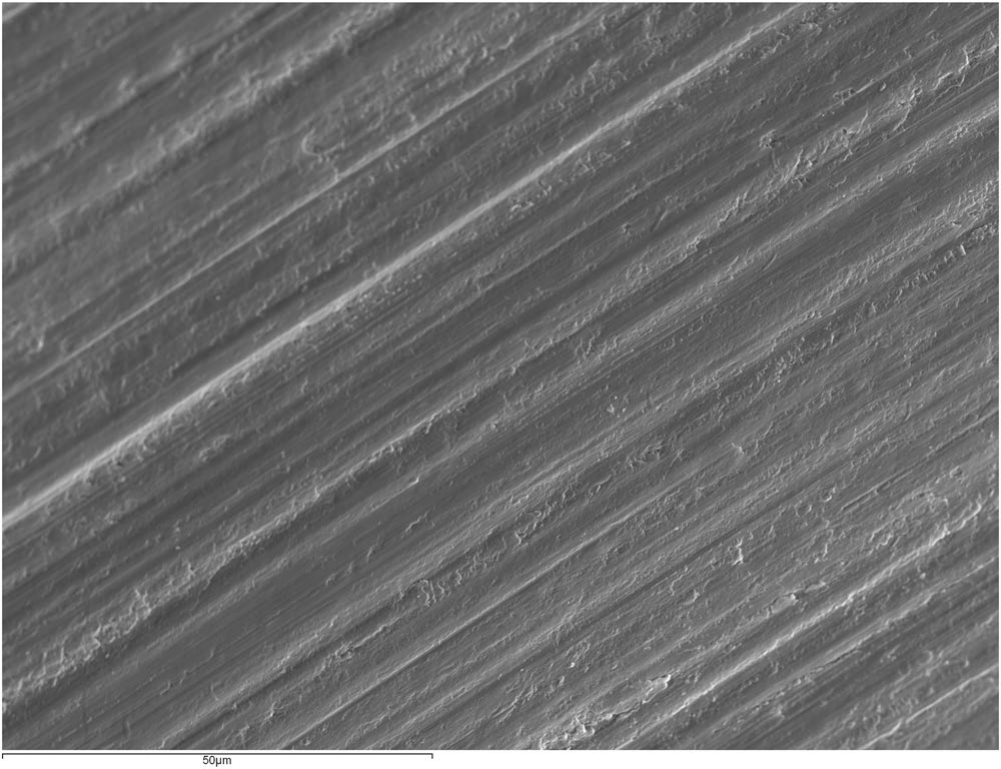
Group 3: Enamel after adhesive removal. SEM micrograph (x1000).

**Figure 4:**
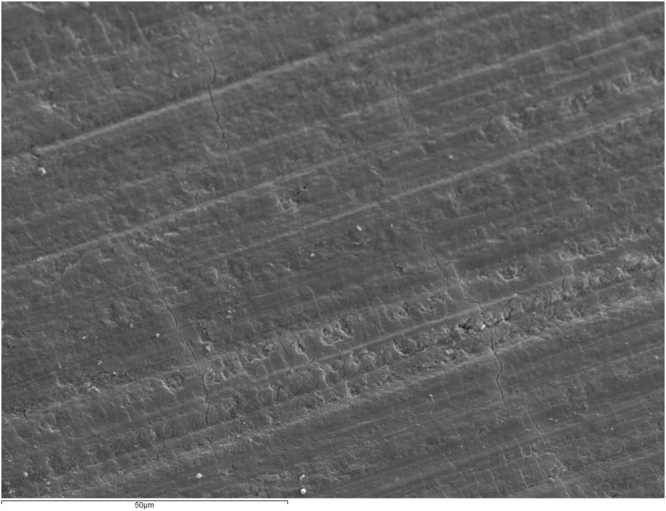
Group 4: Enamel after adhesive removal + DC. SEM micrograph (x1000).

**Figure 5:**
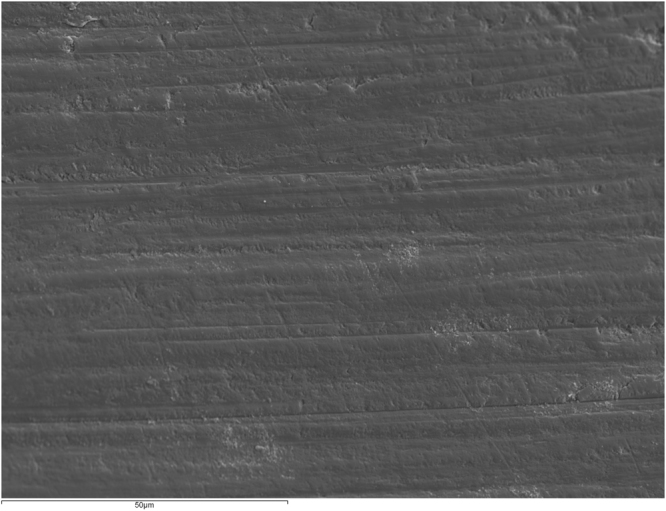
Group 5: Enamel after adhesive removal + Profluorid + DC. SEM micrograph (x1000).

Figure 1 shows an intact enamel surface without demineralization, showing typical morphology of the enamel surface layer. In Group 2 (intact enamel + DC), SEM images reflect a slight demineralization pattern with areas in which the destruction of the centers of prisms predominates (Fig. 2). Figure 3 shows a Group 3 sample (enamel after adhesive removal), with a scratched and faceted surface. In Group 4 (enamel after adhesive removal + DC) the demineralization cycles produced an increase in enamel surface porosity (Fig. 4). Images of a Group 5 sample (enamel after adhesive removal + Profluorid + DC) are similar to Group 3 (enamel after adhesive removal), although they show small isolated areas of early demineralization (Fig. 5).

## Discussion

The aim of this study was to evaluate the elemental content and morphology of enamel subjected to demineralization cycles after bracket debonding, adhesive remnant removal, and fluoride varnish application.

Bovine teeth were used in the study as it is becoming increasingly difficult to obtain extracted human teeth resulting from the increase in preventative dentistry. It has been shown that the composition^17^, microstructure, and orientation of hydroxyapatite crystals^18^ in bovine enamel are similar to that of human teeth.

Like other studies that aim to reproduce clinical situations, the removal of adhesive material remaining on enamel after bracket debonding was performed under a dental operating light and was considered complete when material could no longer be seen on the tooth surfaces and the surface appeared flat^19–21^. A tungsten carbide bur was used for this purpose, polishing the enamel surfaces afterwards with Soft-Lex discs. Many techniques have been proposed for removing remaining adhesive from teeth but none have been universally accepted^22^, as all methods produce some damage to the enamel^7^. The use of tungsten carbide burs removes adhesive more quickly and efficiently than Sof-Lex discs, ultrasonic tools, hand instruments, rubbers or composite burs. However, they roughen the enamel surface, and must be followed by polishing with multi-step Sof-Lex discs^7^. Ryf *et al*.^21^ observed that enamel loss was greater when adhesive was removed by tungsten carbide burs alone, than when followed by polishing.

In the present study, samples underwent three demineralization cycles daily reproducing the ingestion of three main meals per day. Other studies that have evaluated the state of enamel after bracket debonding have not subjected samples to any later demineralization procedure^3,21,23,24^. So these studies do not provide information as to whether the enamel damaged as a result of adhesive removal is or is not more susceptible to the demineralization processes that take place in the oral medium.

The main components of hydroxyapatite are Ca and P, and so these elements were the main objects of study^25^, monitoring Ca and P changes under different experimental conditions.

The results showed a slight decrease in Ca content in Group 2 (enamel after adhesive removal) in comparison with Group 1 (intact enamel). This finding confirms that the weight percentage of Ca and P decrease from the upper surface layers to the more internal layers^25^. Although this decrease in mineral content was not significant, following adhesive removal it was seen to provoke increased demineralization in comparison with the intact enamel group, Ca and P content being significantly higher in Group 2 (intact enamel + DC) than in Group 4 (enamel after adhesive removal + DC). These decreases in Ca and P content also led to changes to the Ca/P ratio, this being significantly higher for intact enamel + DC than enamel after adhesive removal + DC samples. The Ca/P ratio is an indicator of dental tissue mineralization^26^, so these findings affirm that after the removal of adhesive material, the enamel shows a lower degree of mineralization and is more susceptible to further demineralization than intact enamel.

In addition to these changes in the enamel’s elemental content, SEM images also showed that the removal of adhesive alters enamel morphology. In spite of having used a polishing instrument after adhesive remnant removal with a tungsten carbide bur, the images showed increasing enamel roughness by scratching or faceting, a finding that coincides with other studies^7^. The increase in surface roughness has various consequences: on the one hand the adhesion between bacteria and enamel surfaces increases^27^, and on the other, it is difficult to restore shine to the roughened dental surfaces^18^.

In both Group 2 (intact enamel + DC) and Group 4 (enamel after adhesive removal + DC), microscope images showed increases in enamel porosity, with a more defined etching pattern in Group 2 (intact enamel + DC). This is probably due to the fact that in Group 4, resin tags of adhesive material remained between the enamel prisms, as the cleaning procedure does not remove resin that has impregnated the enamel structure^28^.

The present results confirm the need to protect enamel after adhesive removal. The aim of modern dentistry is focused on a prophylactic approach, rather than invasive restoration of carious defects. Although various studies have demonstrated that the application of fluoride varnishes reduces the incidence of caries among the general population^8,9^, as far as we are aware, no other research has investigated fluoride varnish applied as a protective measure against demineralization after orthodontic treatment.

The present results showed that applications of Profluorid protected against demineralization after adhesive removal, as the percentage weight of Ca in Group 5 (Enamel after adhesive removal + Profluorid + DC) was significantly higher than in the Group 4 (Enamel after adhesive removal + DC group), and in turn did not show significant differences in comparison with Group 3 samples (Enamel after adhesive removal). SEM images showed a less porous enamel surface than in Group 4, which were similar to images of Group 3 sample (enamel after adhesive removal).

The presence of F was detected in all the teeth that had received applications of fluoride varnish (Group 5). It could be that this fluoride forms part of calcium fluoride globules (CaF2), as the formation of fluorapatite is a more time-consuming process that requires the presence of fluoride in the mouth for long periods of time^29^. CaF2 complexes precipitate onto the enamel subsurface, and into the inter-prismatic spaces occupied by water and proteins, reducing the enamel’s permeability and so the movement of fluids^30^, constituting an impermeable barrier.

Chersoni *et al*.^30^ demonstrated that even though these complexes (which, in their study, were formed by the use of fluoride toothpaste containing sodium fluoride), could be eliminated mechanically, their removal did not immediately restore permeability by external fluids. In this way the presence of CaF2 could protect enamel and prolong the presence of subsurface salts by delaying their solubilization.

The present study only performed a single bracket debonding procedure, but in the course of orthodontic treatment, teeth may undergo a number of bonding and debonding procedures. Although *in vivo* research is needed to confirm the present findings, the application of a fluoride varnish after debonding could protect dental enamel against the processes of demineralization that take place in the oral medium.

## Conclusions

- The application of Profluorid after bracket debonding and removal of adhesive material remnants from the enamel surface prevents the loss of calcium provoked by demineralization cycles and favors fluoride incorporation into enamel.
- SEM images showed that on enamel surfaces protected by Profluorid, signs of demineralization were not as evident as in those samples that had undergone adhesive remnant removal without fluoride varnish application afterwards.

Although it is necessary to confirm these findings with *in vivo* research, the application of Profluorid could protect dental enamel from demineralization processes after bracket debonding.

## References

[1] Mui B, Rossouw PE, Kulkarni GV (1999). Optimization of a procedure for rebonding dislodged orthodontic brackets. Angle Orthod..

[2] Gaffey PG, Major PW, Glover K, Grace M, Koehler JR (1995). Shear/peel bond strength of repostioned ceramic brackets. Angle Orthod..

[3] Hosein I, Sherriff M, Ireland AJ (2004). Enamel loss during bonding, debonding, and cleanup with the use of a self-etching primer. Am J Orthod Dentofacial Orthop..

[4] Pont HB, Özcan M, Bagis B, Ren Y (2010). Loss of surface enamel after bracket debonding: An *in-vivo* and *ex-vivo* evaluation. Am J Orthod Dentofacial Orthop..

[5] Suliman SN, Trojan TM, Tantbirojn D, Versluis A (2015). Enamel loss following ceramic bracket debonding: A quantitative analysis *in vitro*. Angle Orthod..

[6] Eslamian L (2015). Effect of multiple debonding sequences on shear bond strength of new stainless steel brackets. J Orthod Sci..

[7] Janiszewska-Olszowska J, Szatkiewicz T, Tomkowski R, Tandecka K, Grocholewicz K (2014). Effect of orthodontic debonding and adhesive removal on the enamel - current knowledge and future perspectives - a systematic review. Med Sci Monit..

[8] Greig V, Conway DI (2012). Fluoride varnish was effective at reducing caries on high caries risk school children in rural Brazil. Evid Based Dent..

[9] Bergström EK, Lingström M (2016). Hakeberg. Caries and costs: an evaluation of a school-based fluoride varnish programme for adolescents in a Swedish region. Community Dent Health..

[10] Perrini F, Lombardo L, Arreghini A, Medori S, Siciliani G (2016). Caries prevention during orthodontic treatment: *In vivo* assessment of high-fluoride varnish to prevent white spot lesions. Am J Orthod Dentofacial Orthop..

[11] Du M (2012). Randomized controlled trial on fluoride varnish application for treatment of white spot lesion after fixed orthodontic treatment. Clin Oral Investig..

[12] Singh S, Singh SP, Goyal A, Utreja AK, Jena AK (2016). Effect of various remineralizing agents on the outcome of post-orthodontic white spot lesions (WSLs): a clinical trial. Progress in Orthodontics..

[13] Peng Y (2016). The effect of resin infiltration vs. fluoride varnish in enhancing enamel surface conditions after interproximal reduction. Dent Mater J..

[14] Vicente A, Ortiz Ruiz AJ, González Paz BM, García López J, Bravo González LA (2017). Efficacy of fluoride varnishes for preventing enamel demineralization after interproximal enamel reduction. Qualitative and quantitative evaluation. PLoS One..

[15] Oncag G, Tuncer AV, Tosun YS (2005). Acidic soft drinks effects on the shear bond strength of orthodontic brackets and a scanning electron microscopy evaluation of the enamel. Angle Orthod..

[16] Patil N, Choudhari S, Kulkarni S, Joshi SR (2013). Comparative evaluation of remineralizing potential of three agents on artificially demineralized human enamel: an *in vitro* study. J Conserv. Dent..

[17] Teruel JD, Alcolea A, Hernández A, Ruiz AJ (2015). Comparison of chemical composition of enamel and dentine in human, bovine, porcine and ovine teeth. Arch Oral Biol..

[18] Gomes M (2004). Sistemas adhesivos autograbadores en esmalte: ventajas e inconvenientes. Avances Odontoestomatológicos..

[19] Faria-Júnior ÉM (2015). *In-vivo* evaluation of the surface roughness and morphology of enamel after bracket removal and polishing by different techniques. Am J Orthod Dentofacial Orthop..

[20] Alessandri Bonetti G (2011). Evaluation of enamel surfaces after bracket debonding: An *in-vivo* study with scanning electron microscopy. Am J Orthod Dentofacial Orthop..

[21] Ryf S (2012). Enamel loss and adhesive remnants following bracket removal and various clean-up procedures *in vitro*. Eur J Orthod..

[22] Cochrane NJ, Ratneser S, Reynolds EC (2012). Effect of different orthodontic adhesive removal techniques on sound, demineralized and remineralized enamel. Australian Dental Journal..

[23] Banerjee A, Paolinelis G, Socker M, McDonald F, Watson TF (2008). An *in vitro* investigation of the effectiveness of bioactive glass air-abrasion in the ‘selective’ removal of orthodontic resin adhesive. Eur J Oral Sci..

[24] Al Shamsi AH, Cunningham JL, Lamey PJ, Lynch E (2007). Three-dimensional measurement of residual adhesive and enamel loss on teeth after debonding of orthodontic brackets: an *in-vitro* study. Am J Orthod Dentofacial Orthop..

[25] He B (2011). Mineral densities and elemental content in different layers of healthy human enamel with varying teeth age. Arch Oral Biol..

[26] Paganelli C (2015). Interproximal enamel reduction: an *in vivo* study. Scanning..

[27] Wang C (2015). Effect of enamel morphology on nanoscale adhesion forces of streptococcal bacteria: An AFM study. Scanning..

[28] Zaher AR (2012). Enamel colour changes after debonding using various bonding systems. J Orthod..

[29] Rølla G, Saxegaard E (1990). Critical evaluation of the composition and use of topical fluorides, with emphasis on the role of calcium fluoride in caries inhibition. J Dent Res..

[30] Chersoni S, Bertacci A, Pashley DH (2011). *In vivo* effects of fluoride on enamel permeability. Clin Oral Investig..

